# Prevalence, Influence Factors and Cognitive Characteristics of Mild Cognitive Impairment in Type 2 Diabetes Mellitus

**DOI:** 10.3389/fnagi.2019.00180

**Published:** 2019-07-30

**Authors:** Wei Li, Lin Sun, Guanjun Li, Shifu Xiao

**Affiliations:** ^1^Department of Geriatric Psychiatry, Shanghai Mental Health Center, Shanghai Jiao Tong University School of Medicine, Shanghai, China; ^2^Alzheimer’s Disease and Related Disorders Center, Shanghai Jiao Tong University, Shanghai, China

**Keywords:** T2DM, MCI, Chinese elderly, auditory verbal learning test, cross-section study

## Abstract

**Background**: Type 2 diabetes mellitus (T2DM) is considered as an independent risk factor for mild cognitive impairment (MCI). This study was performed to investigate the prevalence, influencing factors and cognitive characteristics of MCI in elderly patients with T2DM in China.

**Methods**: In the cross-sectional study, we performed cluster random sampling of 3,246 people age 60 years and older across the country. All participants were interviewed and screened for T2DM and MCI. A total of 341 subjects were diagnosed of MCI according to the criteria of Petersen, and a total of 256 subjects were diagnosed of T2DM by using the American Diabetes Association criteria Among the 256 T2DM people, 56 were also diagnosed with MCI. Logistic regression analyses were performed to evaluate risk and protective factor for MCI with T2DM. We also assessed their cognitive function by using the Mini-mental State Examination (MMSE), Montreal Cognitive Assessment (MoCA), Digit span, Associative Learning Test (ALT), Visual Identification Test (VIT), Verbal Fluency (VF), Wechsler Adult Intelligence Scale (WAIS)-III Block Design, WAIS-III picture completion and Auditory Verbal Learning Test (AVLT).

**Results**: Among the 256 T2DM patients, 56 were diagnosed with MCI, and the prevalence of MCI in T2DM was 21.8%. Multivariate logistic regression analyses showed that depression (*p* = 0.002, OR = 6.220, 95% CI: 2.005–19.290) was a risk factor for MCI among T2DM patients, while education (*p* < 0.001, OR = 0.869, 95% CI: 0.805–0.983) was a protective one. All the scores of neuropsychological tests (except for MMSE) in T2DM patients with MCI were lower than those without MCI (*p* < 0.05), but there was no statistical difference (*p* > 0.05) in neuropsychological tests between T2DM-MCI group and No-T2DM-MCI group. Linear regression analysis showed that the drug treatment of diabetes was positively correlated (*t* = 2.263, *p* = 0.025) with the total score of auditory word tests.

**Conclusions**: The present study suggests a high prevalence of MCI among Chinese T2DM patients. Depression is a risk factor for MCI, while education is a protective one. T2DM patients with MCI often show comprehensive cognitive impairment, and the drug treatment of diabetes might help to improve cognitive function.

## Introduction

Type 2 diabetes mellitus (T2DM) is a common metabolic disorder characterized by high blood sugar, insulin resistance, and a relative lack of insulin (Yang et al., 2018). A recent meta-analysis (Yang et al., 2016) pointed out that the overall prevalence (9.1%) of T2DM in China has been increasing since the 1970s, and it increased rapidly with age. In addition to the well-known connection between T2DM and peripheral nervous system disease, diabetes may also cause damage to the central nervous system. What’s more, patients with T2DM generally show worse cognitive performance (Kodl and Seaquist, 2008) and a higher risk of dementia (Arvanitakis et al., 2004; Smith et al., 2010).

Mild cognitive impairment (MCI) is a transitional and possibly modifiable stage between normal cognitive aging and dementia (Petersen, 2011). Compared to healthy individual, people with MCI often have an increased risk to develop dementia (Petersen et al., 1999). Previous studies suggest that T2DM is not only a risk factor for MCI, but also promotes the transformation of MCI into dementia (Cukierman et al., 2005; Li et al., 2016). Therefore, early treatment of T2DM in patients with MCI is critical for improving their prognosis (Cholerton et al., 2016; Xu et al., 2016).

China’s population accounts for 21% of the world population and 1/3 of the Asian population. However, few studies have examined the prevalence of MCI in Chinese T2DM patients [we only found one such study (Gao et al., 2016), but their conclusions are not representative of the country]. So we conduct this national study to explore the prevalence, influence factors and cognitive characteristics of MCI in T2DM among Chinese community elderly.

## Materials and Methods

### Study Population

The China Longitudinal Aging Study (CLAS) is a population-based cohort that is studying randomly sampled participants living in the city communities in China. For a detailed description of the study design, see Xiao et al. (2013). Briefly, 3,246 participants aged 60 years or more from all over the country underwent a baseline examination including a review of their medical history, physical and neurological examinations, laboratory tests, and magnetic resonance imaging (MRI) scans. The recruitment procedure yielded an almost representative sample for the respective communities. The following data, such as name, gender, age, education and daily living information (hobby, dietary preferences, sleeping patterns, smoking history, consumption of tea and alcohol and physical activities) were also collected by standardized questionnaire.

**Figure 1:**
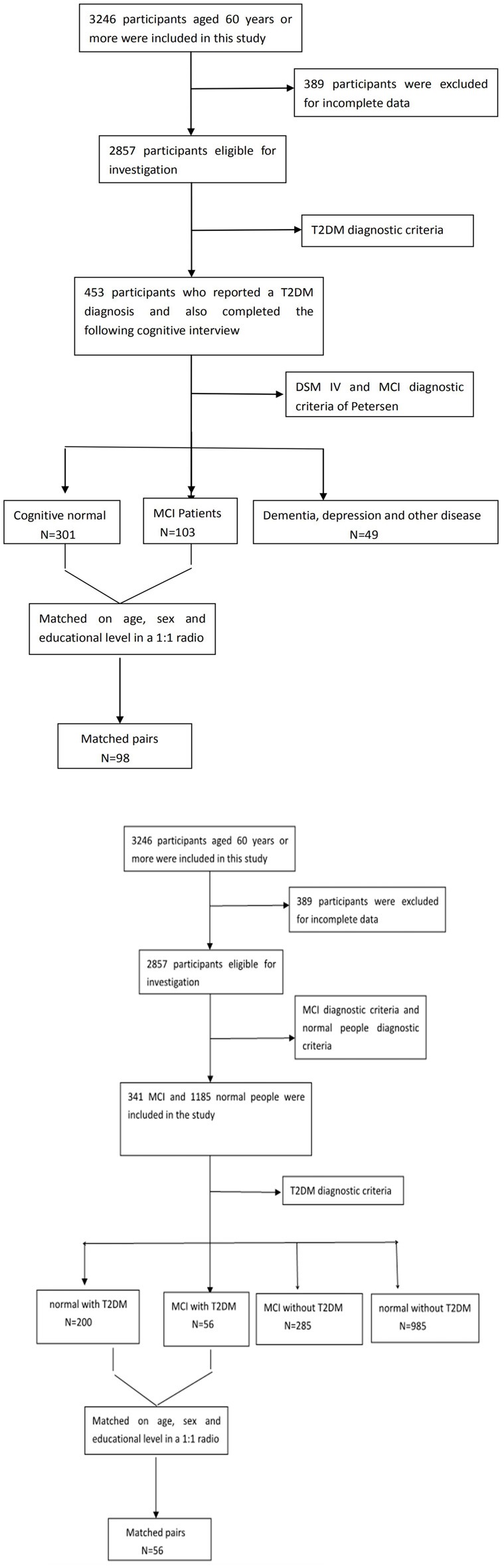
FLow diagram of the study population in the project.

Of the original 3,246 elderly subjects, 389 participants were excluded for the incomplete data; 1,331 people were excluded because they did not meet the diagnostic criteria for MCI or normal cognitive elderly; Among those MCI patients (*n* = 341) and normal people (*n* = 1,185), there were also 256 people (T2DM-MCI, *n* = 56; T2DM-normal, *n* = 200) were diagnosed with diabetes. Figure 1 presents an overview of the study design and sampling procedures.

Ethical approval was obtained from Shanghai Mental Health Centre, and all the participants had signed an informed consent before the study was initiated.

### Measurement of T2DM

Diabetic status was based on self-reported physician’s diagnosis or treatment with insulin and/or oral hypoglycemic agents. Individuals were also considered to have T2DM if their fasting serum glucose level was ≥7.0 mmol/L (126 mg/dl) or their 2-h value in an oral glucose tolerance test was ≥11.1 mmol/L (200 mg/dl; Gao et al., 2015). Finally, 256 participants were considered to have T2DM.

### MCI Diagnostic Criteria

A diagnosis of MCI was based on the following criteria, which were adapted from the MCI diagnostic criteria of Petersen (Portet et al., 2006): (1) memory complaint, preferably corroborated by an informant; (2) objective memory impairment; (3) preservation of general cognitive function; (4) intact activities of daily living; and (5) absence of dementia. Finally, we found that there were 341 MCI patients in the whole population (*n* = 2,857), so the prevalence of MCI in the study population was 11.9%. Of 256 T2DM subjects, 56 subjects were diagnosed with MCI, so the prevalence of MCI among T2DM patients was 21.8% Therefore, we concluded that the prevalence of MCI in diabetic patients was significantly higher than that in normal population (Pearson *x*^2^ = 20.861, *p* < 0.001).

### Cognitive Assessment

Several psychological and psychosocial tests, including the Mini-Mental State Examination (MMSE; O’Bryant et al., 2008), the Montreal Cognitive Assessment (MoCA; Gil et al., 2015), Wechsler Adult Intelligence Scale (WAIS)-III Digit Span (Leung et al., 2011), Auditory Verbal Learning Test (AVLT; Hong et al., 2012), Associative Learning Test (ALT), Visual Identification Test (VIT), Verbal Fluency (VF), WAIS-III Block Design and WAIS-III picture completion were performed to evaluate the cognitive function, such as attention, semantic memory, visuospatial skills, psychomotor speed and executive function, of each subject. The Geriatric Depression Scale (Lin et al., 2016) was also used to exclude depression. All these tests were facilitated by an experienced neuropsychiatrist, and all the subjects were not informed of the study design.

### Statistical Analysis

Statistical analysis was performed by using SPSS version 17.0. The continuous variable and categorical variables were presented as mean ± SD and frequency (%), respectively. All continuous data were tested for normally with the Kolmogorov-Smirnov test. Comparison of continuous data among groups was performed with the single factor analysis of variance (ANOVA) analysis and the results were corrected by using Bonferroni correction. The chi-square test was used for comparison of categorical data. Multiple logistic regression analysis was used for screening the risk and protective factors of MCI. A *P*-value < 0.05 was considered to be significant.

## Results

Figure 1 shows the flow diagram of this study and the demographic variables were shown in Table 1. There were statistical differences (*p* < 0.05) in age, education, systolic blood pressure (SBP), gender, drinking tea, taking exercise, reading, listening to music, suffering the internet, and depression among the T2DM-MCI group, No-T2DM-MCI group and T2DM-Normal group. Then we brought these above variables into multiple regression equation, and the results showed that depression (*p* = 0.002, OR = 6.220, 95% CI: 2.005–19.290) was a risk factor for the development of MCI in patients with diabetes, while education (*p* < 0.001, OR = 0.869, 95% CI: 0.805–0.983) was an important protective factor (Table 2 shows the related results). However, We did not find a significant correlation (*r* = 0.001, *p* = 1.000) between MCI and T2DM through the correlation analysis, suggesting that T2DM might be a adjunctive risk factor for MCI. Next, we compared the cognitive characteristics among the three groups, and 56 pairs of age, sex and education-matched population were selected to participate in the final assessment. We found that there were statistical differences (*p* < 0.05) in the scores of MMSE, MoCA, Digit Span, ALT, VIT, VF, WAIS-III Block Design, WAIS-III picture completion and AVLTs among the three groups (Table 3). Then by using Bonferroni correction, we found that there was no statistical difference (*p* > 0.05) in neuropsychological tests between T2DM-MCI group and No-T2DM-MCI group. But there were statistical differences (*p* < 0.05) in the scores of MoCA, Digit Span, VIT, VF, WAIS-III Block Design, WAIS-III picture completion and AVLTs between T2DM-MCI group and T2DM-Normal group. And there were also statistical differences (*p* < 0.05) in the scores of Digit Span (backward), ALT, VIT, VF, WAIS-III picture completion, and AVLTs between No-T2DM-MCI group and T2DM-Normal group (Table 4). Finally, by using linear regression analysis, we confirmed that the total scores of auditory learning tests were associated with age (*t* = −4.093, *p* < 0.001), education (*t* = 2.392, *p* = 0.018) and treatment of diabetes with drugs (*t* = 2.263, *p* = 0.025; Table 5).

**Table 1:** Demographic, health, and diabetes-related for three groups.

Characteristics	T2DM-MCI (*n* = 56)	No-T2DM-MCI (*n* = 285)	T2DM-Normal (*n* = 200)	*F*	*P*-value
Age, years	74.23 ± 6.93	75.90 ± 7.57	71.12 ± 7.51	23.997	<0.001*
Education, years	4.73 ± 4.94	4.99 ± 4.79	9.00 ± 4.90	42.014	<0.001*
BMI, Kg/m^2^	24.38 ± 2.91	23.51 ± 4.11	24.17 ± 3.20	1.698	0.185
SBP, mmHg	134.91 ± 17.27	129.05 ± 14.73	129.77 ± 15.03	3.537	0.030*
DBP, mmHg	75.12 ± 13.40	76.65 ± 8.12	77.57 ± 8.58	1.758	0.173
Duration of T2DM, years	8.89 ± 7.19	0	8.86 ± 7.35	1.156	0.317
Fasting blood-glucose, mmol/L	12.78 ± 5.35	6.87 ± 4.374	12.09 ± 5.49	0.355	0.701
Tri-glyceride, mmol/L	3.42 ± 1.63	6.96 ± 4.38	3.60 ± 2.26	2.767	0.090
Cholesterol, mmol/L	4.07 ± 4.91	6.95 ± 1.50	7.25 ± 2.21	0.343	0.717
Male (%)	37.5	37.5	50.5	8.678	0.013*
Smoking (%)	24.1	26.3	25.0	0.609	0.738
Drinking alcohol (%)	14.3	18.9	19.0	0.731	0.694
Drinking tea (%)	35.7	36.5	51.0	11.114	0.004*
Eating fish (%)	85.7	86.7	93.0	5.427	0.066
Taking exercise (%)	69.6	55.4	75.0	20.374	<0.001*
Hobby: reading (%)	10.7	11.9	26.5	19.367	<0.001*
Hobby: listening to music (%)	5.4	13.7	21.5	10.478	0.005*
Surfing the internet (%)	0	2.5	7.5	10.312	0.006*
Depression (%)	16.1	9.1	3.5	11.210	0.004*
Sleep disorder (%)	6.8	15.1	14.0	5.644	0.059

^*^p < 0.05.

**Table 2:** Risk and protective factors for MCI in T2DM patients.

Variables	*B*	SE	Wals	*df*	*p*	OR	The 95% CI of OR	
							Lower limit	Upper limit
Education	−0.140	0.039	12.935	1	0.001	0.869	0.805	0.983
Depression	1.828	0.577	10.017	1	0.002	6.220	2.005	19.290

**Table 3:** Results of global cognitive functioning tests and neuropsychological tests in different cognitive domains among three groups.

Neuropsychological test	T2DM-MCI (*n* = 56)	No-T2DM-MCI (*n* = 56)	T2DM-Normal (*n* = 56)	*p*-value
Age, years	74.23 ± 6.93	74.18 ± 6.76	74.68 ± 6.66	0.912
Education, years	4.73 ± 4.94	4.78 ± 5.00	4.59 ± 4.03	0.976
Male (%)	37.5	37.5	37.5	1.000
BMI, kg/m^2^	24.39 ± 2.91	24.11 ± 3.86	24.19 ± 3.38	0.932
Diabetes related information				
Duration of diabetes, years	8.89 ± 7.19	0	7.53 ± 6.90	0.312
Fasting blood glucose, mmol/L	12.78 ± 5.35	0	11.96 ± 6.06	0.491
Dietetic therapy (%)	96.4	0	89.1	0.162
Exercise therapy (%)	69.1	0	69.1	1.000
Drug therapy (%)	98.2	0	85.5	0.016*
Neuropsychological tests				
MMSE	22.13 ± 5.39	22.24 ± 5.09	24.32 ± 4.47	0.035*
MoCA	15.59 ± 6.33	15.91 ± 6.34	18.59 ± 6.05	0.022*
Digit Span (forward)	5.87 ± 2.95	6.68 ± 3.19	7.83 ± 2.77	0.003*
Digit Span (backward)	3.16 ± 2.07	3.43 ± 2.01	4.61 ± 2.83	0.001*
Associative learning test	3.90 ± 3.37	3.03 ± 3.13	5.32 ± 3.52	0.002*
Visual identification test	12.18 ± 4.57	12.16 ± 5.77	15.20 ± 4.72	0.003*
Verbal fluency	17.62 ± 8.19	17.75 ± 7.58	23.00 ± 7.90	0.001*
Wechsler’s filling	5.95 ± 4.04	5.60 ± 3.38	8.22 ± 4.33	0.001*
Wechsler’s building blocks	17.02 ± 9.72	18.15 ± 7.97	22.09 ± 10.50	0.015*
Auditory verbal learning test (T)	41.45 ± 16.96	38.65 ± 21.16	54.89 ± 23.53	0.002*
Auditory verbal learning test 1	3.54 ± 1.96	3.65 ± 2.38	4.67 ± 2.83	0.029*
Auditory verbal learning test 2	5.41 ± 2.46	5.24 ± 2.78	6.56 ± 3.03	0.028*
Auditory verbal learning test 3	6.11 ± 2.76	5.92 ± 3.23	7.26 ± 3.05	0.048*
Auditory verbal learning test 4	6.42 ± 2.90	6.40 ± 3.27	7.98 ± 3.00	0.010*
Auditory verbal learning test 5	7.48 ± 3.01	6.88 ± 3.40	9.11 ± 3.28	0.010*
Auditory verbal learning test 6	4.48 ± 3.48	4.13 ± 4.37	6.13 ± 3.76	0.002*
Auditory verbal learning test 7	2.85 ± 1.69	3.17 ± 1.78	3.65 ± 2.01	0.079
Auditory verbal learning test 8	4.28 ± 3.40	3.73 ± 3.88	5.87 ± 4.20	0.013*

^*^p < 0.05.

**Table 4:** Corrected the results of neuropsychological tests by using Bonferroni correction.

Neuropsychological test	Group-1	Group-2	Average difference	Standard error	*p*	95% confidence interval	
						Lower limit	Upper Limit
MMSE	T2DM-MCI	No-T2DM-MCI	−1.116	0.953	1.000	−2.42	2.19
		T2DM-Normal	−2.196	0.944	0.064	−4.48	0.09
	No-T2DM-MCI	T2DM-Normal	−2.081	0.953	0.091	−4.39	0.22
MOCA	T2DM-MCI	No-T2DM-MCI	−0.032	1.184	1.000	−3.18	2.55
		T2DM-Normal	−3.000	1.179	0.036*	−5.85	−0.15
	No-T2DM-MCI	T2DM-Normal	−2.680	1.184	0.075	−5.55	0.18
Digit Span (forward)	T2DM-MCI	No-T2DM-MCI	−0.807	0.572	0.482	−2.19	0.58
		T2DM-Normal	−1.961	0.570	0.002*	−3.34	−0.58
	No-T2DM-MCI	T2DM-Normal	−1.154	0.575	0.139	−2.54	0.24
Digit Span (backward)	T2DM-MCI	No-T2DM-MCI	−0.273	0.395	1.000	−1.23	0.68
		T2DM-Normal	−1.450	0.394	0.001*	−2.40	−0.50
	No-T2DM-MCI	T2DM-Normal	−1.177	0.399	0.011*	−2.14	−0.21
Associative learning test	T2DM-MCI	No-T2DM-MCI	0.868	0.650	0.550	−0.70	2.44
		T2DM-Normal	−1.428	0.647	0.086	−2.99	0.14
	No-T2DM-MCI	T2DM-Normal	−2.296	0.647	0.002*	−3.86	−0.73
Visual identification test	T2DM-MCI	No-T2DM-MCI	0.025	0.979	1.000	−2.34	2.39
		T2DM-Normal	−3.014	0.979	0.007*	−5.38	−0.64
	No-T2DM-MCI	T2DM-Normal	−3.039	0.997	0.008*	−5.45	−0.63
Verbal fluency	T2DM-MCI	No-T2DM-MCI	−0.135	1.548	1.000	−3.88	3.61
		T2DM-Normal	−5.38	1.540	0.002*	−9.11	−1.66
	No-T2DM-MCI	T2DM-Normal	−5.25	1.540	0.003*	−8.98	−1.52
Wechsler’s filling	T2DM-MCI	No-T2DM-MCI	0.349	0.763	1.000	−1.50	2.19
		T2DM-Normal	−2.277	0.755	0.009*	−4.10	−0.45
	No-T2DM-MCI	T2DM-Normal	−2.626	0.766	0.002*	−4.48	−0.77
Wechsler’s building blocks	T2DM-MCI	No-T2DM-MCI	−1.136	1.825	1.000	−5.55	3.28
		T2DM-Normal	−5.08	1.81	0.017*	−9.45	−0.70
	No-T2DM-MCI	T2DM-Normal	−3.94	1.84	0.102	−8.39	0.52
Auditory verbal learning test	T2DM-MCI	No-T2DM-MCI	2.80	4.46	1.000	−8.03	13.63
		T2DM-Normal	−13.43	4.70	0.015*	−24.86	−2.01
	No-T2DM-MCI	T2DM-Normal	−16.23	4.68	0.002*	−27.60	−4.87

^*^p < 0.05.

**Table 5:** Factors that might affect the score of the auditory vocabulary learning tests.

Group	*B*	Standard error	Standard	*t*	*p*
Age	−1.096	0.268	−0.349	−4.093	<0.001
Education	1.143	0.478	0.204	2.392	0.018
Drug therapy (%)	14.412	6.396	0.178	2.263	0.025

## Discussion

Our report presents the results of a national cross-sectional study that examined the prevalence, risk and protective factor as well as cognitive characteristics of MCI in a population of Chinese elderly patients with Type 2 diabetes. And this study revealed several interesting findings: (1) there was a very high prevalence of MCI among Chinese elderly with Type 2 diabetes; (2) depression was a risk factor for the development of MCI in patients with diabetes, while education was an important protective factor; (3) both overall and specific areas of cognitive function would decline in T2DM patients with MCI, but for patients with MCI, T2DM had no effect on cognitive function; and (4) promptly treating diabetes with drugs might improve the cognitive status of diabetics.

In this study, we found that the prevalence of MCI in Chinese elderly diabetic patients was 21.8%, and it was lower than other data in the worldwide literature. For example, one population-based study suggested that the incidence of MCI in diabetic patients was around 28% (Luchsinger et al., 2007), and another study reported the prevalence of MCI in elders was 31.5% (Gorska-Ciebiada et al., 2014). However, a similar study conducted in China showed that the prevalence of MCI among the elderly population was only 13.5% (Gao et al., 2016). We suspected this disparity might be associated with the MCI diagnostic criteria used. Therefore, it was extremely urgent to unify the diagnostic criteria for MCI.

Like other organs, the brain will also undergo significant structural and functional changes (such as increased neuron loss, decreased synaptic density, declined energy production, disordered synaptic signaling and destructed protein homeostasis) with increasing age (Berchtold et al., 2008; Kohama et al., 2012). And cognitive functioning declines much faster among individuals with MCI than normal cognitive elderly (Cheng and Xiao, 2014). Inconsistent with many other studies (Li et al., 2013; Wang et al., 2017), we did not find that advanced age were a risk factor for MCI, and the difference might be due to the limited sample size.

Depression symptoms occur very commonly in people with MCI and the co-morbidity rate is about 44% (Panza et al., 2010). Previous studies suggest that depression will promote the transformation of MCI into dementia (Barnes and Yaffe, 2011; Van der Mussele et al., 2014). In the present study, we also found that depression was a risk factor for developing MCI. There are several mechanisms that might be used to explain the above phenomenon, first, depression symptoms might increase the burden of amyloid in the cerebral cortex (especially precuneus, parietal, temporal, and occipital regions; Yasuno et al., 2016); second, depression symptoms might alter serotonin metabolism and reduce synaptic plasticity by increasing pro-inflammatory cytokines, such as interleukin-1 and tumor necrosis factor-α (TNF-α; You et al., 2011); third, the concentration of BDNF in the brain of patients with depression is often significantly decreased, which might have a negative impact on the maintenance of neuronal homeostasis and modulation of synaptic plasticity (Lu et al., 2013).

In addition, we also assessed the cognitive function of patients with diabetes. Compared with T2DM patients with normal cognitive function, T2DM patients with MCI showed a decline in both general and specific cognitive fields (Tables s 3, 4). Previous study indicated that type 2 diabetes was associated with impairment of episodic memory and decreased executive function (including working memory, VF, processing speed, cognitive flexibility and cognitive control; McCrimmon et al., 2012; Yaffe et al., 2016), so our conclusions are consistent. By using partial correlation analysis (controlling for age, gender and education), we found that only the total score of AVLT was related to diabetes. And the results of linear regression suggested drug therapy (for diabetes) was associated with better cognitive performance (*t* = 2.263, *p* = 0.025). However, we did not find any negative effect of diabetes on cognitive function in MCI patients. Since this was only a cross-sectional study, further research was needed on the relationship between T2DM and MCI.

There are several limitations to our study. First, this was only a cross-sectional study, and a causal relationship between cognition and T2DM could not be concluded. Second, we did not specifically discuss the relationship between the different subtypes of MCI and cognitive function. Third, longitudinal data from larger samples was needed to verify the above conclusions.

In summary, there is a high prevalence of MCI among Chinese T2DM patients. T2DM patients with MCI are impaired in multiple cognitive fields, but for patients with MCI, T2DM has no effect on cognitive function Depression is a risk factor for developing MCI, while education and use of glucose-lowering medications are protective ones.

## Data Availability

The raw data supporting the conclusions of this manuscript will be made available by the authors, without undue reservation, to any qualified researcher.

## Ethics Statement

Ethical approval was obtained from Shanghai Mental Health Centre, and all the participants had signed an informed consent before the study was initiated.

## Author Contributions

WL wrote this article. LS analyzed the data. GL and SX were project leaders.

## Conflict of Interest Statement

The authors declare that the research was conducted in the absence of any commercial or financial relationships that could be construed as a potential conflict of interest.
